# Dietary Adaptation of Non‐Heme Iron Absorption in Vegans: A Controlled Trial

**DOI:** 10.1002/mnfr.70096

**Published:** 2025-05-05

**Authors:** Miguel López‐Moreno, Isabel Viña, Paula Marrero‐Fernández, Carla Galiana, Gabriele Bertotti, Alberto Roldán‐Ruiz, Marta Garcés‐Rimón

**Affiliations:** ^1^ Diet Planetary Health and Performance Faculty of Health Sciences Universidad Francisco de Vitoria Pozuelo de Alarcón Spain; ^2^ School of Physiotherapy Faculty of Health Sciences Universidad Francisco de Vitoria Pozuelo de Alarcón Spain; ^3^ IVB Wellness Lab Valencia Spain; ^4^ Grupo de Investigación en Biotecnología Alimentaria Universidad Francisco de Vitoria Pozuelo de Alarcón Spain

**Keywords:** hepcidin, iron absorption, non‐heme iron, plant‐based diets, vegan diet

## Abstract

Non‐heme iron, mainly from plant foods, is theoretically less bioavailable than heme iron from animal food, which might increase the risk of iron deficiency in vegans. This study aimed to evaluate acute changes in plasma iron levels following non‐heme iron intake in vegans compared with omnivores and to explore the mechanisms regulating these changes. Twenty‐seven participants (18–30 years old) were divided into vegans and omnivores. After baseline measurements (body composition, blood pressure, and blood biomarkers), the participants consumed 150 g of pistachios. Blood samples were taken at baseline, 120 and 150 min after consumption to measure serum iron. The main outcome was the area under the curve (AUC) of serum iron The AUC for serum iron was significantly higher in vegans (1002.8 ± 143.9 µmol/L/h) compared to omnivores (853 ± 268.2 µmol/L/h) (*p* = 0.04; ES: 0.68). Multivariate regression analysis identified significant associations with hepcidin levels (*β* = −0.5, *p* = 0.03) and basal iron levels in the vegan group. This study demonstrates higher non‐heme iron absorption in vegans compared to omnivores, highlighting the physiological adaptations involved in iron metabolism in plant‐based diets. Larger longitudinal studies are needed to confirm these findings and assess plant‐based diets' long‐term effects on iron metabolism.

## Graphical abstract

This study investigated how non‐heme iron from plant foods is absorbed in vegans compared to omnivores. After consuming pistachios, vegans showed a greater increase in blood iron levels. This may reflect physiological adaptations in iron metabolism, including lower hepcidin levels that promote iron absorption. The findings suggest that plant‐based diets can support efficient iron regulation.

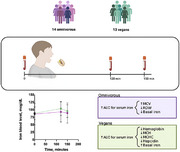

## 1 Introduction

Plant‐based diets have received increasing attention due to their potential benefits for both health and environmental sustainability [1, 2]. These dietary patterns are characterized by prioritizing the consumption of plant‐based foods and include different approaches, such as the vegetarian diet, which excludes meat and fish but often includes dairy products and eggs; and the vegan diet, which excludes all animal products, including meat, dairy products, eggs and honey [3]. Iron is found among the critical nutrients in these diets, which is primarily found as non‐heme iron [4, 5]. As a result, individuals who follow vegetarian or vegan diets might face an increased risk of iron deficiency [6]. However, iron intake is often higher in vegetarians, as legumes, nuts, and whole grains are rich sources of iron. Despite the lower bioavailability of non‐heme iron, this higher intake may help mitigate the risk of iron deficiency [7]. In fact, the European Food Safety Authority (EFSA) does not establish separate dietary reference values for vegetarians, as the overall bioavailability of iron from vegetarian diets is not considered substantially different [8, 9]. This risk is particularly associated when dietary iron requirements are not adequately met, beyond the source of dietary iron, which can lead to iron deficiency anemia, the most advanced stage of iron deficiency [10].

The bioavailability of iron in plant sources is generally lower than animal sources, largely due to the presence of certain compounds, often called “anti‐nutrients,” such as phytates, oxalic acid, and other dietary factors as polyphenols [11, 12]. These compounds can bind to iron, forming complexes that reduce its intestinal absorption [13]. However, the interaction between these compounds and iron metabolism is complex. For instance, phytates have been shown to induce physiological adaptations that enhance intestinal iron absorption, partially compensating their inhibitory effects on bioavailability [14]. One of these adaptations may involve lower levels of hepcidin, a hormone synthesized and secreted by hepatocytes that plays a central role in iron homeostasis.  Hepcidin regulation is particularly significant in states of increased iron demand [15]. Hepcidin is a key regulator of iron homeostasis. It primarily functions by binding to ferroportin, an iron export protein found on the surface of enterocytes and macrophages, triggering its internalization into the cell [16]. Increased iron stores and inflammation enhance hepcidin expression, while reduced iron stores, hypoxia, and hormonal factors (such as testosterone) lower its expression [17]. For example, during pregnancy, plasma hepcidin levels naturally decrease, promoting higher intestinal iron absorption and greater mobilization of stored iron, as reflected by changes in ferritin levels [18]. The absorption mechanisms of heme and non‐heme iron differ, as heme iron is more efficiently absorbed through the heme carrier protein (HCP1), whereas non‐heme iron must be reduced to its ferrous form before being absorbed via the divalent metal transporter 1 (DMT1) [19], In addition, plant‐based foods often contain higher levels of vitamin C, a compound that can increase the absorption of non‐heme iron by 8%–20% [20]. The regulation of these transporters, along with hepcidin's role in modulating ferroportin, represents a key mechanism likely influenced by dietary habits and the resulting availability of iron in the diet.

Previous studies have evaluated iron absorption in individuals following diets with low iron bioavailability [21], such as lacto‐ovo‐vegetarian diets [22]. However, in these cases, the participants were not previously vegetarian or vegan, making it difficult to identify potential long‐term physiological adaptations. To the authors' knowledge, to date no clinical trial has evaluated acute changes in plasma iron levels following the intake of non‐heme iron in individuals who were already vegan, compared to omnivores. The main objective of this study was to evaluate acute changes in plasma iron levels in response to non‐heme iron intake in vegans compared to omnivores and to explore the physiological mechanisms underlying their regulation.

## 2 Participants and Methods

Participants aged 18–30 years were recruited during September and October 2024 through public announcements and flyers located on the university campus (Universidad Francisco de Vitoria, Madrid). The sample size was estimated with the G*Power software (3.1.9.7; Heinrich Heine University of Düsseldorf, Germany), assuming a medium effect size, a statistical power of 90% and an *α* error probability of 0.05, which indicated that at least 26 participants needed to be included [21]. Vegan participants were required to have adhered to a diet free of animal‐derived foods, including meat, fish, dairy, eggs, and other animal‐based products, over the past 6 months. In contrast, omnivorous participants were required to have consistently followed a diet that included both animal and plant‐based foods during the same period. Adherence was verified through dietary records and participant interviews to ensure consistency in following either the vegan or omnivorous diet. Additional inclusion criteria applied to both groups were non‐smoking status and low alcohol consumption (less than 10 g of alcohol per day). Exclusion criteria included pregnancy or breastfeeding, blood donation within the past 6 months, use of medications or supplements known to interfere with iron absorption in the past 6 months (e.g., antacids, proton pump inhibitors, calcium or zinc supplements, antibiotics), consumption of iron supplements in the past 6 months, any gastrointestinal condition that could affect dietary iron absorption (including celiac disease, inflammatory bowel disease [IBD], gastric bypass surgery, and chronic gastritis), or any known allergy to soy or nuts. Participant eligibility was assessed through an ad hoc pre‐participation questionnaire and screening. Prior to enrollment, eligible participants were thoroughly informed about the potential risks and possible discomforts associated with the research protocol. Subsequently, they were asked to provide written informed consent to participate in the study. The study protocol and design were approved from the Ethics Committee of the University Francisco de Vitoria (32/2024) and fully adhered to the ethical principles outlined in the 1964 Declaration of Helsinki, including its most recent revision (2013).

### 2.1 Study Protocol

The study consisted of a single visit to the Exercise Physiology Research Laboratory at Francisco de Vitoria University. Initially, participants completed a validated 93‐item food frequency questionnaire to confirm their dietary patterns and ensure compliance with the study's inclusion criteria [23]. Thereafter, blood pressure measurements, body composition analysis, and baseline blood sample collection were performed. Body mass, body fat mass, body fat percentage, and musculoskeletal mass were measured using electrical bioimpedance analysis (Inbody Co., Ltd., Seoul, Korea) with manufacturer‐specific predictive equations. To ensure accurate and reliable measurements, participants adhered to specific conditions, including overnight fasting, refraining from strenuous activity, and avoiding alcohol and caffeine intake for 24 h before testing. All measurements were conducted in a controlled environment. Systolic blood pressure (SBP), diastolic blood pressure (DBP), and resting heart rate were then measured in triplicate using a digital sphygmomanometer (Omron, Japan). Anthropometric assessments by bioimpedance followed previously described standards [24]. Body height was measured with the participant standing fully erect, feet together, and head aligned in the Frankfort plane, and arms hanging naturally, and was recorded with millimeter accuracy (0.1 cm). Body mass index (BMI) was calculated using the formula weight (kg)/[height (m)^2^]. A basal blood sample was also collected before the administration of the test meal.

Following these measurements, participants consumed 150 g of pistachios (approximately 79 g of edible portion) as a dietary source of non‐heme iron [25]. The edible portion was calculated by subtracting the remaining pistachios after consumption from the total amount served to the participants. The iron content of the test meal was calculated to be 5.7 mg (± 0.6 mg/100 g), previously quantified by a certified laboratory (Laboratorio KUDAM S.L., Valencia). Subsequently, blood samples were collected 120 and 150 min after ingestion of the test meal. During this period, participants remained at rest in the laboratory and were not allowed to consume any food or drink other than water. Participants were also instructed to avoid physical activity before and during the testing session to ensure standardized conditions for measuring changes in plasma iron levels.

### 2.2 Blood Sample Analysis

The analysis of the biomarkers was carried out by authorized personnel at a certified clinical laboratory in Madrid, Spain (Megalab S.L.), using fasting venous blood samples. The following baseline parameters were determined: erythrocytes (10^6^/µL), hemoglobin (g/dL), hematocrit (%), mean corpuscular volume (MCV, fL), mean corpuscular hemoglobin (MCH, pg), mean corpuscular hemoglobin concentration (MCHC, g/dL), red cell distribution width (RDW, %), and ferritin (ng/mL). Iron levels (µg/dL) were measured at 0, 120, and 150 min. Blood samples for hepcidin and soluble transferrin receptor (sTfR) measurements were centrifuged, and the resulting serum was aliquoted into microcentrifuge tubes and stored at −20°C until analysis. Hepcidin and sTfR levels were quantified using enzyme‐linked immunosorbent assay (ELISA) kits obtained from Elabscience Biotechnology Co., Ltd. (Wuhan, China). The hepcidin kit had a measurement range of 0.78–50 ng/mL, with a sensitivity of 0.32 ng/mL, and a coefficient of variation under 10%. Similarly, the sTfR kit had a measurement range of 6.25–400 ng/mL, a sensitivity of 3.75 ng/mL, and a coefficient of variation of less than 10%. All procedures were performed according to the manufacturer's instructions. Measurement of hepcidin and sTfR were performed spectrophotometrically at a wavelength of 450 nm using a multimode plate reader (Biotek HT Sinergy, Vermont, VT, USA). This procedure was carried out in the Biochemistry Laboratory of the Francisco de Vitoria University.

### 2.3 Statistical Analysis

The statistical analysis was performed using GraphPad Prism 6 (GraphPad Software, Inc.) and IBM Statistical Package for the Social Sciences (SPSS) version 22.0 (IBM, Chicago, IL, USA). Continuous variables are presented as mean ± standard deviation (SD). The normality of the data for each variable was assessed using the Shapiro‐Wilk test. Baseline characteristics were compared by Student's *t* test or Mann–Whitney *U* test, depending on the distribution of the variables. Univariate regression models were used to examine the potential influence of clinical, anthropometric, dietary, and biochemical variables on the area under the curve (AUC) of serum iron. Variables that showed a significant relationship were included in a multivariate regression model, adjusted for potential confounders such as age, sex, and BMI. The results were presented as the unstandardized regression coefficient (*B*) in the univariate model, the standardized regression coefficient (*β*) in the multivariate model, together with the corresponding 95% confidence interval (CI), and the change in the *R*‐squared coefficient of determination after inclusion of each variable. The models were calculated separately for the entire group of participants and for the vegan and omnivore subgroups.

Effect size (ES) was assessed using Cohen's *d* for normally distributed variables, with the following interpretation criteria: trivial (0–0.19), small (0.20–0.49), medium (0.50–0.79), and large (≥0.80) [26]. For non‐normally distributed variables, the effect size was assessed by the probability of superiority (PS), with the following criteria: no effect (PS < 0.56), small effect (PS ≥ 0.56), medium effect (PS ≥ 0.64), and large effect (PS ≥ 0.71) [27]. Statistical significance was set at *p* ≤ 0.05.

## 3 Results

Of the 65 individuals who underwent the initial screening, 38 did not meet the inclusion criteria. Exclusions were mainly due to scheduling conflicts that prevented attendance at the tests (*n* = 10), following a vegetarian diet (*n* = 8), taking iron supplements (*n* = 6), having gastrointestinal conditions that could affect dietary iron absorption (*n* = 6), undergoing a blood transfusion in the past 6 months (*n* = 4), being smokers (*n* = 2), and having a peanut allergy (*n* = 2). Consequently, a total of 27 subjects were enrolled in the study. Regarding sex, there was a similar distribution between the individuals in the omnivorous group (71% female) and those in the vegan group (69% female). Table 1 presents the age, anthropometric data, and laboratory measurements of the study participants at baseline. No significant differences were observed between the groups for any of the baseline analyzed parameters, except for hepcidin levels, which were significantly lower in the vegan participants (*p* = 0.02; PS = 0.58).

**TABLE 1:** Baseline characteristics of participants including demographic, anthropometric, hematological, and biochemical parameters.

	Omnivorous (*n* = 14; 10 F, 4 M)	Vegan (*n* = 13; 9 F, 4 M)	*p*
Age	26.6 ± 3.70	28.0 ± 4.69	0.30
Weight (kg)	66.9 ± 15.5	66.1 ± 9.86	0.88
BMI	22.7 ± 2.65	22.3 ±2.30	0.68
Musculoskeletal mass (kg)	29.6 ± 8.95	30.6 ± 5.70	0.73
Body fat (kg)	13.7 ± 3.93	11.2 ± 4.65	0.15
Body fat (%)	20.9 ± 5.7	16.9 ± 6.24	0.09
SBP (mmHg)	126.9 ± 19.8	120.3 ± 14.2	0.33
DBP (mmHg)	73.9 ± 9.36	70.4 ± 11.2	0.39
Heart rate (bpm)	67.7 ± 15.5	61.4 ± 5.5	0.17
Erythrocytes (10^6^/µL)	4.59 ± 0.31	4.67 ± 0.39	0.56
Hemoglobin (g/dL)	13.8 ± 1.01	14.3 ± 1.32	0.29
Hematocrit (%)	41.0 ± 2.57	42.0 ± 3.68	0.44
MCV (fL)	89.2 ± 3.78	90.2 ± 8.56	0.06
MCH (pg)	30.1 ± 1.63	30.7 ± 3.02	0.39
MCHC (g/dL)	33.6 ± 0.63	34.0 ± 0.57	0.10
RDW (%)	13.5 ± 0.73	13.1 ± 0.89	0.24
Ferritin (ng/mL)	70.4 ± 61.2	99.2 ± 102.7	0.35
Hepcidin (ng/mL)	24.3 ± 9.0	13.6 ± 8.0	0.02
sTRF (mg/L)	2.0 ± 2.5	3.8 ± 1.5	0.24

*Note*: Mean ± standard deviation (SD).

Abbreviations: BMI, body mass index; DBP, diastolic blood pressure; F, female; M, male; MCH, mean corpuscular hemoglobin; MCHC, mean corpuscular hemoglobin concentration; MCV, mean corpuscular volume; RDW, red cell distribution width; SBP, systolic blood pressure; sTfR, soluble transferrin receptor.

*Significant differences using Student's *t*‐test, *p* < 0.05.

**Significant differences using the Mann–Whitney *U* test, *p* < 0.05.

**FIGURE 1:**
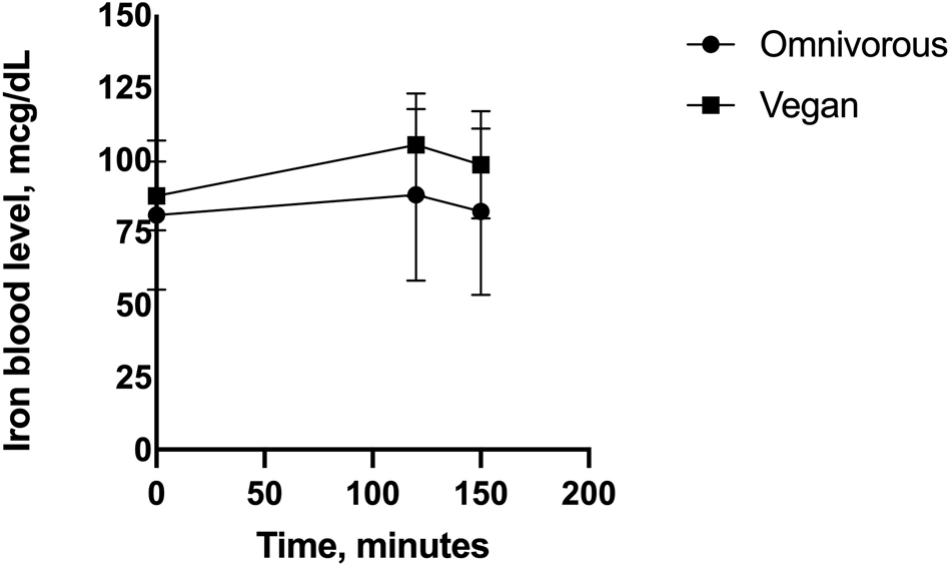
Iron blood levels after the consumption of a test meal in omnivorous (*n* = 13) and vegans (*n* = 14) groups. The Values representing biological replicates are expressed as means ± standard deviation (SD). Significant differences using Student's *t*‐test, *p* < 0.05.

Figure 1 illustrates the serum iron curves for the vegan and omnivorous groups at baseline and after the test meal. Baseline iron levels, as well as levels measured at 120 and 150 min after consumption of the test meal, did not differ between groups (*p* > 0.05) (Table 2). The increase in iron levels at 120 min after the test meal consumption was higher in vegan participants (17.3 µg/dL ± 9.9) compared to omnivorous participants (7.4 µg/dL ± 6.8) (*p* = 0.03; ES: 0.39). However, no differences between groups were observed in the increase of iron levels 150 min after the test meal consumption (*p* > 0.05). The AUC for serum iron was significantly higher in vegan participants (1002.8 ± 143.9 µmol/L/h) compared to omnivorous participants (853 ± 268.2 µmol/L/h) (*p* = 0.04; ES: 0.68).

**TABLE 2:** Baseline and post‐meal iron levels, iron changes, and AUC for serum iron.

	Omnivorous (*n* = 14) Mean ± SD	Vegan (*n* = 13) Mean ± SD	*p*	ES
Basal iron	80.8 ± 25.8	87.4 ± 11.9	0.39	0.32
Iron (120 min)	87.8 ± 29.6	105.0 ± 17.8	0.08	0.70
Δ Iron (120 min)	7.4 ± 6.8	17.3 ± 9.9	0.03*	0.39
Iron (150 min)	82.1 ± 28.6	98.2 ± 18.5	0.09	0.66
Δ Iron (150 min)	5.4 ± 6.7	6.8 ± 7.9	0.40	0.16
iAUC (µmol/L/h)	853.9 ± 268.2	1002.8 ± 143.9	0.04*	0.68

*Note*: Mean (µg/dL) ± standard deviation (SD).

Abbreviations: ES, effect size; iAUC, area under curve for serum iron.

*Significant differences using Student's *t*‐test, *p* < 0.05.

**Significant differences using the Mann–Whitney *U* test, *p* < 0.05.

In univariate regression analyses including all participants, a higher AUC for serum iron values was associated with lower levels of hemoglobin (*B* = −64.5, *p* = 0.01), MCHC (*B* = −264.3, *p* = 0.02), RDW (*B* = −24.7, *p* = 0.01), ferritin (*B* = −38.5, *p* = 0.02), and basal iron (*B* = −9.7, *p* < 0.01). In omnivorous participants, the AUC for serum iron values was related to MCV (*B* = 46.5, *p* < 0.01), lower levels of RDW (*B* = −57.2, *p* < 0.01), and basal iron (*B* = −9.3, *p* < 0.01). Among vegan participants, the AUC for serum iron values was inversely correlated with the levels of hemoglobin (*B* = −95.4, *p* < 0.01), MCH (*B* = −116.4, *p* = 0.03), MCHC (*B* = −163.3, *p* = 0.02), hepdicin (*B* = −13.9, *p* = 0.03), and positively correlated with basal iron (*B* = 8.8, *p* < 0.01) (Table 3).

**TABLE 3:** Univariate regression of AUC for serum iron with demographics and iron status markers in the whole group (*n* = 27), omnivorous (*n* = 13), and vegans (*n* = 14) subgroups.

	All participants		Omnivorous		Vegans	
	*B* (95% CI)	*p*	*B* (95% CI)	*p*	*B* (95% CI)	*p*
Age	12.1 (−7.07; 31.3)	0.20	12.2 (−31.0; 55.4)	0.55	−1.2 (−21.6; 19.1)	0.89
Sex	150.4 (−22.0; 322.9)	0.08	243.1 (−68.9; 555.0)	0.11	−24.1 (−212.1; 163.8)	0.78
BMI	−4.5 (−42.9; 33.2)	0.81	13.7 (−47.0; 74.5)	0.63	−23.5 (−61.8; 14.8)	0.20
Red blood cells (10^6^/µL)	10.2 (−254.3; 274.8)	0.93	−187.6 (−695.6; 320.6)	0.43	94.2 (−138.4; 326.7)	0.39
Hemoglobin (g/dL)	−64.5 (−139.3; −10.3)	**0.01**	−23.2 (−93.8; 47.5)	0.48	−95.4 (−146.5; −55.7)	**<0.01**
Hematocrit (%)	18.7 (−9.8; 47.3)	0.19	24.2 (−37.3; 85.7)	0.41	9.7 (−15.4; 34.8)	0.41
MCV (fL)	8.7 (−5.2; 22.8)	0.21	46.5 (14.9; 78.0)	**<0.01**	−0.4 (−11.5; 10.8)	0.94
MCH (pg)	−130.7 (−160.1; 37.5)	0.09	−2.48 (−34.0; 29.0)	0.88	−116.4 (−184.0; −48.9)	**0.03**
MCHC (g/dL)	−264.3 (−463.7; −64.7)	**0.02**	−41.2 (−206.4; 123.8)	0.59	−163.3 (−296.9; −29.7)	**0.02**
RDW (%)	−24.7 (−27.8; −2.8)	**0.01**	−57.2 (−97.4; −10.8)	**<0.01**	0.10 (−106.6; 106.8)	0.99
Ferritin (ng/mL)	−38.5 (−60.8; −15.7)	**0.02**	−35.5 (−29.5; 6.0)	0.20	−17.7 (−19.0; 17.5)	0.69
Hepcidin (ng/mL)	−3.78 (−12.7; 3.1)	0.36	6.70 (−12.4; 25.8)	0.46	−13.9 (−21.5; −4.7)	**0.03**
sTfR (mg/L)	26.3 (−14.2; 66.8)	0.19	12.7 (−29.5; 94.8)	0.28	31.7 (−13.8; 47.2)	0.30
Basal iron (µg/dL)	−9.7 (−11.9; −7.5)	**<0.01**	−9.3 (−11.7; −6.9)	**<0.01**	8.8 (−14.3; −3.4)	**<0.01**

Abbreviations: B, regression coefficient; CI, confidence interval; BMI, body mass index; MCH, mean corpuscular hemoglobin; MCHC, mean corpuscular hemoglobin concentration; MCV, mean corpuscular volume; RDW, red cell distribution width; sTfR, soluble transferrin receptor.

Bold values indicate statistically significant results (*p* < 0.05).

In the multivariate regression model including all participants, the AUC for serum iron values was significantly associated with higher values of hemoglobin (*β* = −0.3, *p* = 0.04) and basal iron (*β* = −0.9, *p* = 0.01) (Table 4). Among omnivorous participants, the AUC for serum iron values was positively related to basal iron (*β* = 1.1, *p* = 0.01). In vegans, the AUC for serum iron values was positively associated with hepcidin values (*β* = −0.5, *p* = 0.03) and inversely associated with basal iron (*β* = −0.6, *p* = 0.01). The *R*‐squared was 80.2% for the entire sample, 36.6% for omnivorous participants, and 85.6% for vegans.

**TABLE 4:** Multivariate regression of AUC for serum iron values with iron status markers in the whole group (*n* = 27), omnivorous (*n* = 13), and vegans (*n* = 14) subgroups (adjusted for age, sex, and BMI).

	All participants		Omnivorous		Vegans	
	*β*	*p*	*β*	*p*	*β*	*p*
Hemoglobin (g/dL)	−0.3	**0.04**	0.3	0.31	0.2	0.62
MCV (fL)	5.4	0.31	2.7	0.60	0.1	0.93
MCH (pg)	−6.6	0.27	−3.9	0.53	0.3	0.78
MCHC (g/dL)	1.6	0.25	1.4	0.55	0.4	0.56
RDW (%)	−0.9	0.59	−0.005	0.98	0.7	0.64
Ferritin (ng/mL)	−0.3	0.14	−0.08	0.92	−0.4	0.42
Hepcidin (ng/mL)	−0.1	0.30	0.2	0.80	−0.5	**0.03**
Basal iron (µg/dL)	−0.9	**0.01**	1.1	**0.01**	−0.6	**0.01**
*R* ^2^ (%)	80.2		36.6		85.6	

Abbreviations: MCH, mean corpuscular hemoglobin; MCHC, mean corpuscular hemoglobin concentration; MCV, mean corpuscular volume; RDW, red cell distribution width.

Bold values indicate statistically significant results (p < 0.05).

## 4 Discussion

The present study evaluated changes in plasma iron levels after acute non‐heme iron intake in vegans compared to omnivorous individuals, as well as predictive factors influencing this response. The increase in serum iron at 120 min and the change in the AUC for serum iron were significantly higher in vegans, suggesting a potentially enhanced acute iron absorption response in this group. Among the main markers of iron status, basal hemoglobin and iron levels predicted the AUC for serum iron in the overall sample, whereas baseline hepcidin and iron levels influenced the AUC results for serum iron in vegan participants.

Our study provides insights into the dietary adaptation of non‐heme iron absorption in vegan participants compared to omnivorous. The AUC for serum iron after acute non‐heme iron intake was significantly higher in participants following a vegan diet, suggesting greater efficiency of dietary iron absorption compared with those following an omnivorous diet. Previous studies have evaluated the adaptation of iron absorption in response to diets with higher versus lower iron bioavailability. Dietary iron bioavailability tends to adjust to either increase or decrease to maintain iron homeostasis. For instance, in men with normal iron stores, the proportion of non‐heme iron absorption decreased when consuming a diet with high iron bioavailability and increased when following a diet with low iron bioavailability [28]. In women with suboptimal iron stores, a higher dietary non‐heme iron absorption was observed after 10 weeks on a diet high in phytates—present in plant‐based foods—even though they acutely inhibit dietary iron absorption [21]. However, lower non‐heme iron absorption has also been reported in healthy individuals following a vegetarian diet compared to an omnivorous diet for 8 weeks [22]. This may be due to the fact that potential adaptations to non‐heme iron intake in a diet with low iron bioavailability are not immediately apparent, and their effects may take longer to become evident, depending on the duration and specific composition of the diet, as well as the individual's iron status [29].

In our study, participants following a vegan diet had adhered to this dietary pattern for at least the past 6 months, which suggests a sufficient period for these potential adaptations to become established, thereby promoting a better absorption of non‐heme iron. Thus, it can be hypothesized that, as dietary iron bioavailability gradually influences body iron stores, the efficiency of iron absorption subsequently adjusts to compensate for these changes, and the body aims to maintain its homeostatic balance or biological set point for iron levels [30, 31]. This is corroborated by the present study, in which participants following a vegan diet showed no significant differences compared to those on an omnivorous diet in several markers of iron status, such as plasma hemoglobin, hematocrit, MCV, and ferritin levels.

The greater increase in serum iron in vegans suggests that vegans may exhibit physiological mechanisms that compensate for the lower bioavailability of dietary iron. This could be attributed to the upregulation of iron absorption pathways due to sustained dietary exposure to plant‐based sources of non‐heme iron. The regression analyses provided further context for these findings. In vegans, the significant associations between the AUC for serum iron and hepcidin, as well as basal iron levels, suggest key underlying adaptive mechanisms. Hepcidin is a central regulator of iron homeostasis, controlling iron absorption by binding to ferroportin, the iron exporter located in basolateral membrane of enterocyte, and reducing its activity by internalization and degradation (ubiquititation) [32, 33]. This regulation helps balance iron levels by limiting the release of iron from stores and decreasing absorption from the diet [34]. In contrast, a vegan diet, which is rich in non‐heme iron, may benefit from reduced hepcidin levels, as this could facilitate greater iron absorption from the intestines to compensate for dietary limitations. In fact, lower hepcidin levels have also been observed in children following a vegetarian diet when compared to omnivores [35]. Therefore, lower hepcidin could enhance the mobilization of iron stores or improve the efficiency of non‐heme iron uptake, thus helping to maintain adequate iron homeostasis despite the lower bioavailability of dietary iron. In vegetarian children, a slight increase in sTfR has been observed, suggesting homeostatic changes that enhance iron utilization and absorption in diets with less bioavailable iron [36]. Increased sTfR indicates higher expression of TfR in erythrocyte precursors, promoting more efficient iron uptake even with lower plasma iron levels [37]. However, in our study we did not observe different levels of sTfR, which rules out this mechanism as one of the factors involved in the increased absorption reported in vegan participants. Additionally, the gut microbiota may also play a role in iron absorption regulation [38]. Indeed, it can adjust its iron metabolism based on iron availability through the ferric‐uptake regulator protein (Fur), conserved in both Gram‐negative and Gram‐positive bacteria, which influences the expression of genes involved in iron transport [39]. This regulatory process could also contribute to improved iron absorption in individuals with plant‐based diets.

These findings may have implications for setting iron recommendations and requirements in vegans, given the differences in iron absorption efficiency compared to omnivores. This suggests that the theoretical bioavailability rates used to establish these recommendations may vary between populations and that the bioavailability of non‐heme iron could be underestimated in populations following plant‐based diets, with significant variations observed among studies [40, 41]. Heme iron is absorbed directly through the intestinal wall, independently of the body's iron requirements [42, 43]. In contrast, non‐heme iron absorption is more tightly regulated, with increasing uptake when the body requires iron—an adaptive mechanism that helps prevent iron overload [44]. Although iron is essential for health, excessive levels can have deleterious effects by inducing oxidative stress via the Fenton reaction, which generates highly reactive free radicals [45, 46]. This process has been associated with cellular damage, lipid peroxidation, and ferroptosis—an iron‐dependent cell death mechanism potentially implicated in neurological disorders [47]. Furthermore, elevated heme iron intake has been linked to an increased risk of certain cancers, including colon and breast cancer, underscoring the importance of achieving a proper balance in dietary iron management [48, 49, 50, 51].

One of the main strengths of our study is the controlled design, which allows a direct comparison of dietary iron adaptation between vegans and omnivores. Additionally, the use of AUC for serum iron as an integrative measure of postprandial iron response enhances the robustness of our findings. Moreover, the vegan participants in our study had been following a plant‐based diet for at least the past 6 months, ensuring that any observed adaptations in iron absorption and metabolism are not merely short‐term responses, but represent sustained physiological adjustments. However, the present study has several limitations. The relatively small sample size and the lack of long‐term follow‐up may limit the generalizability of the results. Future research involving larger cohorts is recommended to enhance the robustness of the findings. Furthermore, although our study provides valuable insights into on the mechanisms of iron absorption, the influence of individual variability in dietary patterns, genetic polymorphisms affecting hepcidin expression, and gut microbiota composition on iron absorption requires further investigation [52, 53]. Additionally, longer‐term studies are needed to assess how these physiological adaptations may influence iron stores over time. These factors could play an important role in iron homeostasis, and a more comprehensive understanding could provide a broader perspective on how different dietary groups, including vegans and omnivores, adapt to variations in iron bioavailability. Additionally, the limited blood sampling time points may have restricted the ability to fully capture the absorption curve, and future studies could benefit from more frequent sampling to better assess the kinetics of iron absorption.

## 5 Conclusion

This study highlights the dynamic interaction between diet and iron absorption, as vegan participants demonstrate enhanced non‐heme iron absorption as reflected by higher AUC values for serum iron. These results contribute to a greater understanding of dietary adaptations to the plant‐based diet and their implications for iron homeostasis and overall health. Future studies with larger, longitudinal cohorts are needed to confirm these findings and explore the long‐term effects of plant‐based diets on iron metabolism, and investigate the role of gut microbiota in iron absorption. Furthermore, it would be valuable to investigate whether enhanced absorption mechanisms persist over different durations of plant‐based diets, as this could yield valuable insights in this matter. Including assessments of iron balance, such as iron excretion rates, would also provide a more comprehensive understanding of iron homeostasis in plant‐based diets.

## Conflicts of Interest

IV is employed by *IVB Wellness Lab*. The other authors declare that there is no conflict of interest about the content of the present study.

## Peer Review

The peer review history for this article is available at https://publons.com/publon/10.1002/mnfr.70096.

## Data Availability

The data that support the findings of this study are available from the corresponding author upon reasonable request.
